# Protective Effects of Korean Red Ginseng against Alcohol-Induced Fatty Liver in Rats

**DOI:** 10.3390/molecules200611604

**Published:** 2015-06-23

**Authors:** Hyo Jin Lee, Hyang Mok Ok, Oran Kwon

**Affiliations:** Department of Nutritional Science and Food Management, Ewha Women’s University, 52 Ewhayeodae-gil, Seodaemun-gu, Seoul 120-750, Korea; E-Mails: lhj264@hanmail.net (H.J.L.); holytreeok@gmail.com (H.M.O.)

**Keywords:** Korean red ginseng, alcoholic fatty liver, adipose tissue, AMP-activated protein kinase

## Abstract

The present study tested the hypothesis that Korean red ginseng (KRG) provides a protective effect against alcoholic fatty liver. Male Sprague-Dawley rats were divided into four groups and fed a modified Lieber-DeCarli diet containing 5% (*w*/*v*) alcohol or an isocaloric amount of dextrin-maltose for the controls for 6 weeks: normal control (CON), alcohol control (ET), and ET treated with 125 or 250 mg/kg body weight/day of KRG (RGL or RGH, respectively). Compared with the CON group, the ET group exhibited a significant increase in triglycerides, total cholesterol and the presence of lipid droplets in the liver, and a decrease in fat mass, which were all attenuated by KRG supplementation in adose-dependent manner. The mitigation was accompanied by AMP-activated protein kinase (AMPK) signaling pathways in the liver and adipose tissue. In addition, suppression in the alcohol-induced changes of adipose adipokine mRNA expression was also observed in KRG supplementation group. These findings suggest that KRG may have the potential to ameliorate alcoholic fatty liver by suppressing inappropriate lysis of adipose tissue and preventing unnecessary *de novo* lipogenesis in the liver, which are mediated by AMPK signaling pathways. A mechanism for an interplay between the two organs is still needed to be examined with further assays.

## 1. Introduction

Alcoholic liver disease remains one of the most common etiologies of liver disease and has become a major cause of morbidity and mortality worldwide [1]. Hepatic steatosis (alcoholic fatty liver), defined as excess lipid accumulation in the cytoplasm of hepatocytes, is the most common and earliest consequence of chronic and excessive alcohol consumption and is regarded as a significant risk factor for hepatic fibrosis and cirrhosis [2]. In recent years, considerable progress has been made toward understanding the mechanisms underlying the development of alcoholic fatty liver. The major pathogenetic factors include increased *de novo* lipogenesis, impaired mitochondrial fatty acid β-oxidation, and decreased very low-density lipoprotein (VLDL) secretion in the liver [3]. However, the mechanism of alcoholic fatty liver formation is very complex; thus, controversies remain in the literature [4].

The accumulating evidence has suggested that adipose tissue dysfunction might impact hepatic lipid metabolism [5,6]. Recently, Zhong *et al.* [7], by utilizing deuterium-labeled triglycerides, revealed a direct link between adipose triglyceride loss and hepatic triglyceride gain in alcohol-fed mice. Dysregulation of lipid homeostasis was also evidenced in clinical studies, which presented a lower mass of fat during the development of alcoholic fatty liver [8]. Considering that adipose tissue plays an important role as a major metabolic buffering system for lipid metabolic homeostasis [9,10], modification of alcohol-induced dysfunction in adipose tissue might be an important target for development of functional foods to prevent alcoholic fatty liver [9,11].

Ginseng, the root of Panax ginseng Meyer, has been traditionally employed in Asia for manipulating alcohol intoxication [12]. Modern pharmacological studies have revealed that individual bioactive components in ginseng, such as ginsenoside Rg3 [13,14] and Re [15] attenuate hepatic lipid accumulation *in vitro*. Several recent publications have reported that Korean Red Ginseng (KRG) has protective effects against body weight increase [16] and liver damage [17] in animal models. More recently, Bang *et al*. [18] evaluated the efficacy of KRG in mouse models of alcoholic liver disease, with a focus on immunologic capacity. However, the effects of KRG on alcohol-induced dysregulation of lipid homeostasis and the underlying mechanism(s) responsible for it remain un addressed. In the present study, we tested a hypothesis that KRG may have the potential to protect against alcoholic fatty liver. To test this hypothesis, we determined lipid profiles in plasma and the liver and made a histological observation in the liver of rats fed Lieber-DeCarli Diets containing 5% (*w*/*v*) alcohol. Furthermore, to explore the underlying mechanism, we compared the changes in AMPK signaling pathways in the liver and adipose tissue.

## 2. Results and Discussion

### 2.1. Effects of KRG on Body Weight and Food Intake

**Figure 1:**
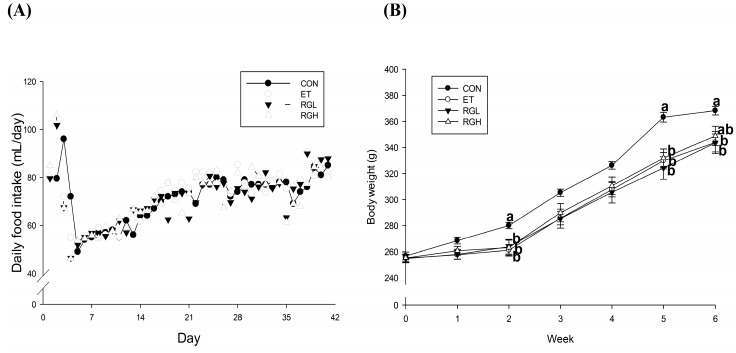
Changes in daily food intake (**A**) and body weight (**B**) in rats fed alcohol and KRG for 6 weeks: (●) CON, normal control; (○) ET, ethanol control; (▼) RGL, ET + KRG 125 mg/kg body weight; (∆) RGH, ET + KRG 250 mg/kg body weight. Alcohol was provided at 5% of the Lieber-DeCarli liquid diet, and KRG was administered daily by gavage for the same time period. Data are expressed as the means ± SE (*n* = 10/group). The values with the different letters are significantly different based on Duncan’s multiple range tests, *p* < 0.05.

Body weight and food intake were compared in Figure 1. Daily food intake was controlled so that the rats in different groups received as nearly as possible the same amount of food for the entire experimental period (Figure 1A). Nonetheless, the ET group presented a significantly lower body weight compared with the CON group. KRG supplementation did not cause any body weight changes (Figure 1B).

### 2.2. Effects of KRG on Lipid Profiles in Plasma and the Liver

**Figure 2:**
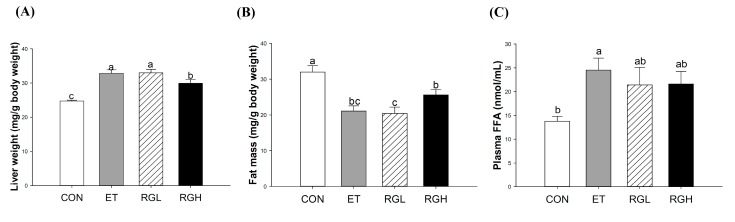

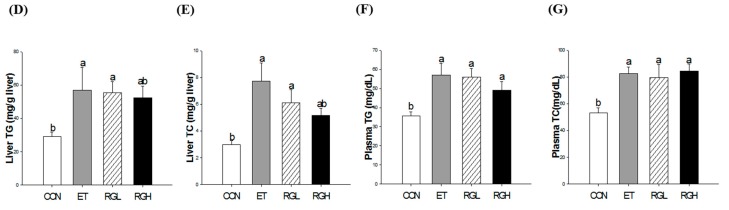
Lipid profiles in rats fed alcohol and KRG for 6 weeks. (**A**) Liver weight; (**B**) fat mass (epididymal, perirenal, and mesenteric); (**C**) plasma free fatty acids; (**D**) hepatic triglycerides; (**E**) hepatic total cholesterol; (**F**) plasma triglyceride; and (**G**) plasma total cholesterol. CON, normal control; ET, alcohol control; RGL, alcohol + 125 mg/kg KRG; RGH, alcohol + 250 mg/kg KRG. Alcohol was provided at 5% of the Lieber-DeCarli liquid diet, and KRG was administered daily by gavage for the same time period. Data are expressed as the means ± SE (*n* = 10/group). The values with the different letters are significantly different based on Duncan’s multiple range tests, *p =* 0.05.

Six-week alcohol feeding resulted in significant increases in the liver-to-body weight ratio, which was attenuated by concomitant administration of KRG (*p =* 0.0001) (Figure 2A). In contrast, the fat mass (epididymal, perirenal, and mesenteric) was significantly decreased in the ET group, but remained high in rats fed high level of KRG (*p =* 0.0001) (Figure 2B). The plasma FFA level increased significantly in the ET group, but remained low in the groups with co-administered KRG (*p* = 0.04) (Figure 2C). Significant increases in hepatic triglycerides (TG) (Figure 2D) and total cholesterol (TC) (Figure 2E) were observed in the ET group, which were suppressed significantly by concomitant administration of KRG (*p* = 0.0333 and 0.001, respectively). However, no changes were observed in plasma TG (Figure 2F) and TC (Figure 2G).

### 2.3. Effects of KRG on Histological Changes in the Liver

**Figure 3:**
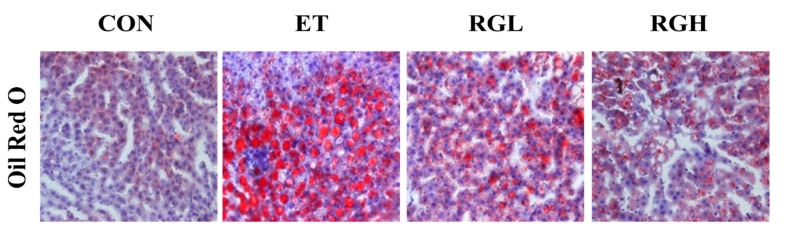

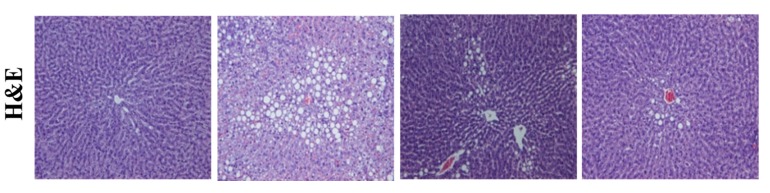
Confirmation of hepatic fatty liver in rats fed alcohol and KRG for 6 weeks. Hepatic histology: Oil Red O stained (upper panel) and H & E stained (lower panel). CON, normal control; ET, alcohol control; RGL, alcohol + 125 mg/kg KRG; RGH, alcohol + 250 mg/kg KRG. Alcohol was provided at 5% of the Lieber-DeCarli liquid diet, and KRG was administered daily by gavage for the same time period. Data are expressed as the means ± SE (*n =* 10/group).

The Oil Red O (ORO) and Hematoxylin and Eosin (H & E) staining techniques were employed to examine lipid droplet accumulation and histological changes in the liver. A massive accumulation of lipid droplets was found in the ET group. Normal cells exhibited lobular architectures, but cells in the ET group exhibited panlobular mixed micro/macro vesicular steatosis and focal clusters of inflammatory cells with the associated necrosis. In contrast, simultaneous administration of KRG meaningfully prevented the above pathologic changes (Figure 3).

### 2.4. Effects KRG on AMPK Signaling Pathways in the Liver and Adipose Tissue 

AMPK phosphorylation and its downstream effector genes and enzymes were determined in the liver and adipose tissue by utilizing q-PCR and western blotting analysis.

**Figure 4:**
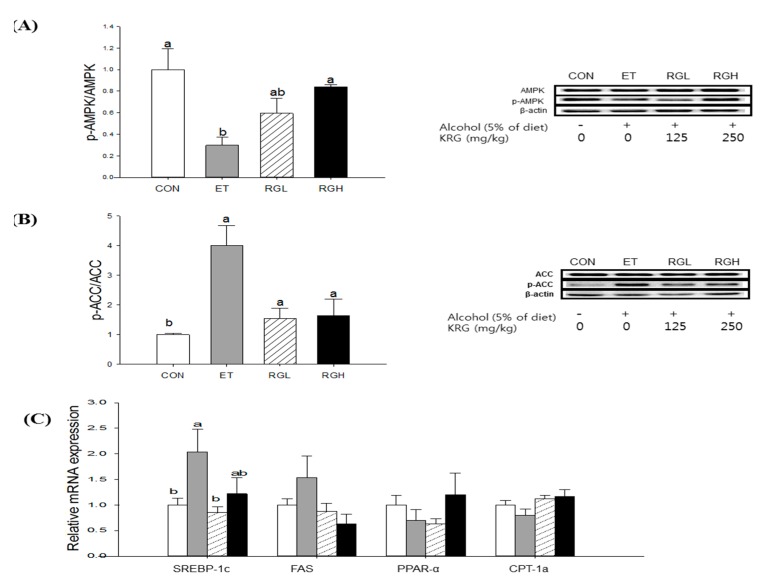
Protein phosphorylation and mRNA expression in the liver of rats fed alcohol and KRG for 6 weeks. (**A**) AMPK phosphorylation; (**B**) ACC phosphorylation; and (**C**) mRNA expressions of SREBP-1c, FAS, PPAR-α and CPT-1a. CON, normal control; ET, alcohol control; RGL, alcohol + 125 mg/kg KRG; RGH, alcohol + 250 mg/kg KRG. Alcohol was provided at 5% of the Lieber-DeCarli liquid diet, and KRG was administered daily by gavage for the same time period. Data are expressed as the means ± SE (*n =* 10/group). The values with the different letters are significantly different based on Duncan’s multiple range tests, *p =* 0.05.

In the liver, co-administration of KRG reversed the alcohol-induced suppression of AMPK phosphorylation in a dose-dependent manner (*p* = 0.0194) (Figure 4A). Subsequently, alcohol-induced increases in ACC phosphorylation (*p* = 0.0081) (Figure 4B) and SREBP-1c mRNA expression (*p* = 0.0047) (Figure 4C) were suppressed significantly by KRG administration. However, no further changes were found in FAS, PPAR-α, and CPT-1a mRNA expression.

**Figure 5:**
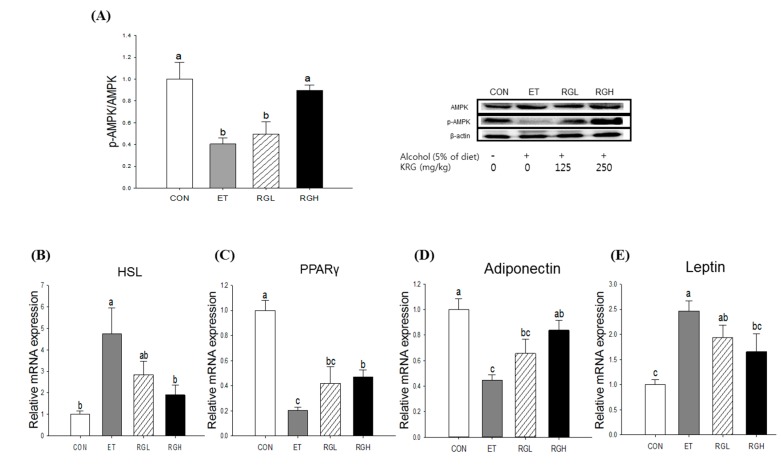
Protein phosphorylation and mRNA expression in adipose tissue of rats fed alcohol and KRG for 6 weeks. (**A**) AMPK phosphorylation; (**B**) HSL mRNA expression; (**C**) PPAR-γ mRNA expression; (**D**) Adiponectin mRNA expression, and (**E**) Leptin mRNA expression. CON, normal control; ET, alcohol control; RGL, alcohol + 125 mg/kg KRG; RGH, alcohol + 250 mg/kg KRG. Alcohol was provided at 5% of the Lieber-DeCarli liquid diet, and KRG was administered daily by gavage for the same time period. Data are expressed as the means ± SE (*n =* 10/group). The values with the different letters are significantly different based on Duncan’s multiple range tests, *p =* 0.05.

We also found that KRG administration reversed the alcohol-induced inhibition of AMPK phosphorylation in adipose tissue (*p* = 0.008) (Figure 5A). Subsequently, alcohol-induced increases in HSL (*p* = 0.0076) (Figure 5B) and PPARγ (*p =* 0.0001) (Figure 5C) mRNA expressions were obviously attenuated dose-dependently when KRG was co-administered. In contrast, alcohol-induced suppression of adiponectin mRNA was restored by KRG administration (*p* = 0.0003) (Figure 5D), and the reverse was found in leptin mRNA expression in adipose tissue (*p* = 0.0015) (Figure 5E).

### 2.5. Discussion

Alcoholic fatty liver is a uniform response by the liver toward alcohol that can progress, resulting in increasingly severe liver diseases. Despite the growing body of literature on the functionality of KRG [19,20], the preventive effects of ginseng against alcohol-induced fatty liver *in vivo* and the underlying mechanism responsible for it remain unclear. Therefore, in the present study, we tested the hypothesis that KRG has a preventive effect against alcoholic fatty liver, focusing on the role of lipid metabolic homeostasis at the liver-adipose tissue axis. An increasing number of studies have indicated that chronic alcohol exposure for weeks stimulates adipose tissue lipolysis, leading to an increased fatty acid influx to the liver. In line with the previous studies, the current study demonstrated an alcohol-induced disruption of lipid homeostasis as manifested by a dramatic decrease in fat mass, an increase in plasma free fatty acids, and an increase in hepatic lipid droplets. However, these changes were all reversed by KRG administration.

In this study, we adopted a paired-feeding protocol employing a Lieber-DeCarli liquid diet because it was important to maintain identical caloric intake among the groups to avoid a confounding caused by an energy imbalance. In addition, the Lieber-DeCarli liquid diet is a nutritionally competent diet that mimics chronic drinking patterns in humans and allows for the administration of high amounts of alcohol and the induction of significant liver injury [21]. Moreover, during the first three days of the experiment, alcohol consumption was gradually increased from 1.25% to 5% (*w*/*v*) to avoid a sudden decrease of food intake. Consequently, an addition of alcohol into totally liquid, nutritionally adequate diets at the level of 5% (*w*/*v*) for 6 weeks was found to be appropriate for studying the early stage of alcoholic fatty liver, which did not progress to hepatitis, as evidenced by H&E and ORO staining and inflammatory cytokine levels in the liver. Rats did not drink enough alcohol when given drinking water [22]. Other studies have also documented that the Lieber-deCarli model is associated with neither inflammation nor fibrosis [23,24].

Among the various regulators, we focused on the AMPK signaling pathway as the underlying mechanism. AMPK is a heterotrimeric protein kinase that is ubiquitously expressed and plays an important role for cellular energy homeostasis as it senses the AMP: ATP ratio [25]. In the liver, AMPK phosphorylation leads to a concomitant stimulation of catabolic pathways and inhibition of biosynthetic pathways [26]. In the current study, we observed that KRG administration restored alcohol-induced decreased AMPK phosphorylation, leading to a significant decrease in ACC phosphorylation and SREBP-1c mRNA expression. The critical role of SREBP-1c in alcoholic fatty liver has been supported in intra-gastric infusion model [27] and SREBP-1c knockout mice [28]. In contrast, in adipose tissue, we found that KRG administration restored alcohol-induced inhibition of AMPK phosphorylation, leading to attenuating triglyceride lipolysis through down-regulating HSL and up-regulating PPARγ. Adipose HSL is a key enzyme involved in triglyceride lipolysis and FFA efflux [29], and PPARγ plays an important role during adipogenesis [30]. Taken together, the results obtained in this study suggest that the protection of KRG against alcoholic fatty liver is, at least partially, attributed to suppression of inappropriate lysis of adipose tissue and prevention of unnecessary *de novo* lipogenesis in the liver, both mediated by AMPK signaling pathways.

Adipose tissue can secrete adipokines such as adiponectin and leptin in response to various metabolic signals [31]. The pivotal role of adiponectin in the development of alcoholic fatty liver has been suggested by a number of independent studies [32,33]. In this study, we demonstrated that KRG administration also influenced alcohol-induced changes in adiponectin and leptin mRNA expression in adipose tissue. In adipose tissue, leptin is negatively associated with adiponectin level [34]. Although some studies reported that moderate alcohol ingestion has an inhibitory effect on leptin secretion [35] resulting in an increased appetite [36], in the present study, leptin mRNA expression was significantly increased by alcohol administration, probably leading to a decrease in appetite and body fat mass. We found that these changes were reversed by KRG supplementation. However, it is worth noting that we did not measure either direct changes of leptin secretion or the expression of adipokine receptors in the liver. This is a limitation of this study and future studies are needed to elucidate the molecular mechanisms of KRG on the interplay between adipose tissue and the liver.

## 3. Experimental Section

### 3.1. Materials

**Figure 6:**
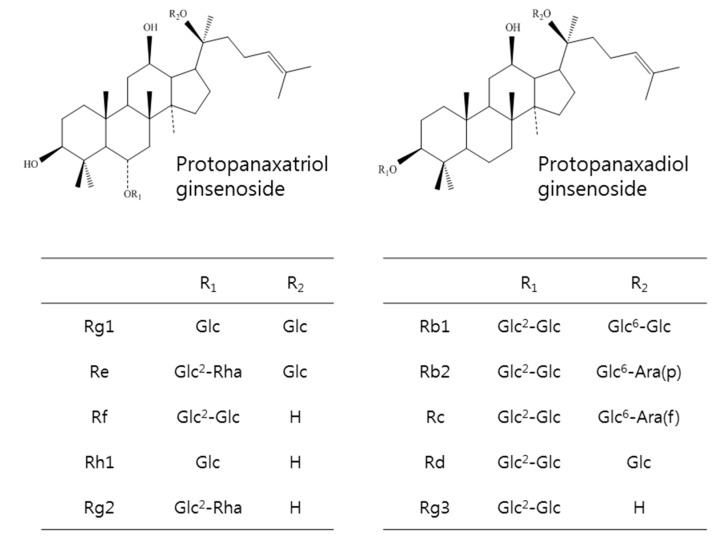
Chemical structures of ginsenosides in KRG.

KRG was provided by the Korean Ginseng Corporation (Daejeon, Chung-cheong-do, Korea). Briefly, roots of 6-year-old red ginseng, Panax ginseng Meyer, were prepared to contain approximately 27 mg/g of total ginsenosides following hot water extraction and concentration. A high performance liquid chromatography analysis of the chemical signature revealed that major ginsenosides and their contents were Rb1 (10.32 mg), Rc (4.11 mg), Rb2 (3.95 mg), Rg1 (2.58 mg), Rg3 (1.62 mg), Rf (1.29 mg), Rd (1.07 mg), Rg2 (1.00 mg), Rh1 (0.71 mg), and Re (0.11 mg) per gram of KRG (Figure 6). 

### 3.2. Animals and Diets

Seven-week-old male Sprague-Dawley rats were purchased from Jung-Ang Lab Animal Inc. (Seoul, Korea) and acclimatized to the laboratory setting (22.0 ± 2.0 °C, 12 h light/dark cycles, 55% ± 5% humidity) with free access to water and food (Samyang Co, Incheon, Korea) for a week before the experiment. The rats (220–230 g) were randomly divided into four groups based on their body weight (*n =* 10 per group): normal control (CON), alcohol control (ET), and ET treated with KRG at 125 or 250 mg/kg (RGL or RGH, respectively). The level of KRG was determined based on our preliminary studies. The rats were fed a Liber-DeCarli diet, containing either 5% (*w*/*v*) alcohol (ranges from 1.25% for 0–2 days to 5%) or an isocaloric amount of dextrin-maltose in water for the controls, providing 36% of the energy as alcohol for 6 weeks [37] (Table 1). The Lieber-Decarli liquid diets were purchased from Dyets, Inc. (Bethlehem, PA, USA). KRG was dissolved in distilled water and administered by gavage at the same time each day during the entire period of the experiment. Body weight was measured weekly and dietary intake was recorded daily. At the end of the experiment, the rats were fasted overnight, euthanized by CO_2_ inhalation, and exsanguinated through cardiac puncture. The blood sample was centrifuged (1500× *g*, 4 °C, 10 min) to separate the plasma and stored at −80 °C before analysis. To examine histological changes, parts of the livers were preserved with phosphate-buffered formalin. The rest of the liver and adipose tissues were frozen at −80 °C until analysis. The experimental protocol was approved by the Institutional Animal Care and Use Committee of Ewha Womans University (Seoul, Korea), and all experimental procedures were conducted in compliance with the guidelines of Ewha Womans University for the care and use of laboratory animals.

### 3.3. Biochemical Assays in Plasma and the Liver

For determination of lipid profiles in the liver, frozen liver tissue was homogenized in a chloroform: methanol mixture (2:1, *v*/*v*) and centrifuged at 3000 rpm for 20 min. The chloroform phase was aspirated and filtered utilizing sodium sulfate. The filtered chloroform phase was evaporated, and the lipid residue was collected. The residue was dissolved in phosphate-buffered saline containing 1% Triton X-100. 

The plasma free fatty acid (FFA) concentration was measured utilizing an enzymatic assay kit (BioVision, Palo Alto, CA, USA). TC and TG were analyzed in the plasma and liver utilizing enzymatic assay kits (Asan, Seoul, Korea).

### 3.4. Histopathology Analysis

The formalin-fixed liver tissues were dehydrated in a 70%–100% gradient of ethyl alcohol, dealcoholized in xylene, embedded in paraffin, and sectioned (cut into 5-µm thickness). The liver sections were deparaffinized in xylene, rehydrated in a reverse-gradient series of ethyl alcohol, and stained with H & E and ORO. The slides were dehydrated, dealcoholized, mounted utilizing Canada balsam, and assessed for inflammation and tissue damage utilizing an Olympus microscope (Olympus, Tokyo, Japan).

### 3.5. Total RNA Isolation and Quantitative Real-Time PCR Analyses

The total RNA was extracted from the liver and adipose tissue utilizing TRIzol (Invitrogen, Carlsbad, CA, USA). The RNA concentration and quality were measured utilizing BioSpec-nano (Shimadzu, Kyoto, Japan). cDNA was constructed utilizing a High Capacity RNA-to-cDNA kit (Applied Biosystems). A quantitative real-time polymerase chain reaction (RT-PCR) was performed utilizing the TaqMan method and a Step-One-Plus RT-PCR System (Applied Biosystems, Foster City, CA, USA). The primer set for the rat target genes was sterol regulatory element binding protein-1c (SREBP-1c) [Scap; Rn01446560_m1], peroxisome proliferator-activated receptor α (PPARα) [Ppara; Rn00566193_m1], carnitine palmitoyl transferase-1 (CPT-1) [Cpt1a; Rn00580702_m1], fatty acid synthase (FAS) [Fasn; Rn00569117_m1], hormone sensitive lipase (HSL) [Lipe; Rn00563444_m1], PPARγ [Pparg; Rn00440945_m1], leptin [Lep; Rn00565158_m1], adiponectin [Adipoq; Rn00595250_m1], and β-actin [Actb; Rn00667869_m1]. The reactive amounts of these mRNAs were normalized to the amount of β-actin, and the relative amounts of all of the RNA were calculated employing the comparative CT method [38]. All of the data are expressed relative to control values.

### 3.6. Western Blot Analysis

The proteins for the liver and adipose tissue were extracted with lysis buffer (Intron Biotech, Seoul, Korea) and quantified by the Bradford method. The total protein was resolved utilizing 10% SDS-PAGE and transferred to polyvinylidene difluoride membranes (Bio-Rad, Hercules, CA, USA). After blocking with 3% bovine serum albumin (BSA) and 5% skim milk in Tween/Tris-buffered saline (TBS) at room temperature for 90 min, the membranes were washed three times in TBS and probed overnight at 4 °C with rabbit anti-AMP-activated protein kinase (AMPK) (1:1000, Cell Signaling Technology, Danvers, MA, USA), rabbit anti-p-AMPK (1:1000, Cell Signaling Technology), rabbit anti-acetyl-coenzyme A carboxylase (ACC) (1:1000, Cell Signaling Technology), rabbit anti-p-ACC(1:1000, Cell Signaling Technology), and mouse anti-β-actin (1:1000, Santa Cruz Biotechnology, Santa Cruz, CA, USA) in 5% BSA + TBS. The immunoreactive antigens were detected after incubation at room temperature for 120 min with horseradish peroxidase-labeled anti-rabbit or anti-mouse/rabbit IgG (1:2000, Santa Cruz Biotechnology) in 5% BSA + TBS. The immunoreactive bands were quantified utilizing a ChemiDoc XRS + System with the Image Lab software (Bio-Rad). To ensure equal loading, the relative target protein levels were normalized relative to β-actin.

### 3.7. Statistical Analysis

All of the results are presented as the means ± standard errors (SEs). The statistical analyses were performed employing Statistical Analysis Systems software version 9.3 (SAS institute Cary, NY, USA). Data were analyzed utilizing a one-way analysis of variance (ANOVA) with post-hoc Duncan’s multiple range tests. A value of *p =* 0.05 was considered statistically significant.

## 4. Conclusions

In this study, we demonstrated the protective effects of KRG against alcoholic fatty liver, focusing on the involvement of the AMPK signaling pathway at the liver and adipose tissue. Although large uncertainties remain in a complicated signaling network, our findings provide solid evidence that supports a liver-adipose tissue interaction by KRG administration for modulating alcoholic fatty liver and provide a rationale for further evaluation in humans.

## Abbreviations

AMPK: AMP-activated protein kinase
CPT: carnitine palmitoyl transferase
FFA: free fatty acid
HSL: hormone sensitive lipase
KRG: Korean red ginseng
PPAR: peroxisome proliferator-activated receptor
SREBP: sterol regulatory element-binding protein
VLDL: very low-density lipoprotein
WAT: white adipose tissue
HSL: hormone sensitive lipase
